# HIV Testing and Care in Canadian Aboriginal Youth: A community based mixed methods study

**DOI:** 10.1186/1471-2334-8-132

**Published:** 2008-10-07

**Authors:** Judy E Mill, Randy C Jackson, Catherine A Worthington, Chris P Archibald, Tom Wong, Ted Myers, Tracey Prentice, Susan Sommerfeldt

**Affiliations:** 1 Faculty of Nursing, University of Alberta, 7-50 University Terrace, Edmonton, Alberta, Canada; 2 Canadian Aboriginal AIDS Network, Ottawa, Ontario, Canada; 3 Faculty of Social Work, University of Calgary, Calgary, Alberta, Canada; 4 Public Health Agency of Canada, Ottawa, Ontario, Canada; 5 Faculty of Medicine, University of Toronto, Toronto, Ontario, Canada

## Abstract

**Background:**

HIV infection is a serious concern in the Canadian Aboriginal population, particularly among youth; however, there is limited attention to this issue in research literature. The purpose of this national study was to explore HIV testing and care decisions of Canadian Aboriginal youth.

**Methods:**

A community-based mixed-method design incorporating the Aboriginal research principles of Ownership, Control, Access and Possession (OCAP) was used. Data were collected through surveys (n = 413) and qualitative interviews (n = 28). Eleven community-based organizations including urban Aboriginal AIDS service organizations and health and friendship centres in seven provinces and one territory assisted with the recruitment of youth (15 to 30 years).

**Results:**

Average age of survey participants was 21.5 years (median = 21.0 years) and qualitative interview participants was 24.4 years (median = 24.0). Fifty-one percent of the survey respondents (210 of 413 youth) and 25 of 28 interview participants had been tested for HIV. The most common reason to seek testing was having sex without a condom (43.6%) or pregnancy (35.4%) while common reasons for not testing were the perception of being low HIV risk (45.3%) or not having had sex with an infected person (34.5%). Among interviewees, a contributing reason for not testing was feeling invulnerable. Most surveyed youth tested in the community in which they lived (86.5%) and 34.1% visited a physician for the test. The majority of surveyed youth (60.0%) had tested once or twice in the previous 2 years, however, about one-quarter had tested more than twice. Among the 26 surveyed youth who reported that they were HIV-positive, 6 (23.1%) had AIDS at the time of diagnosis. Delays in care-seeking after diagnosis varied from a few months to seven years from time of test.

**Conclusion:**

It is encouraging that many youth who had tested for HIV did so based on a realistic self-assessment of HIV risk behaviours; however, for others, a feeling of invulnerability was a barrier to testing. For those who tested positive, there was often a delay in accessing health services.

## Background

The overall HIV infection rate among Aboriginal persons in Canada has been estimated to be 2.8 times higher than among non-Aboriginal persons [1]. In 2006 Aboriginal persons accounted for 24.4% of the reported AIDS cases for which ethnicity was known [2], however, only 3.8% of the Canadian population report Aboriginal ancestry [3]. Almost half of the Canadian Aboriginal population are 24 years or younger with a median age of 27 years (Statistics Canada). Coupled with this demographic, Aboriginal youth are over-represented in the HIV epidemiological data. Canadian Aboriginal persons are infected with HIV at a younger age and at a higher rate than non-Aboriginals [2]. High rates of teenage pregnancy [4], inconsistent condom use [4,5], high rates of sexually transmitted infections (STIs) [6], along with feelings of invulnerability, contribute to the susceptibility of Aboriginal youth to HIV infection.

Traditionally, HIV prevention approaches have focused on uninfected populations. More recently, reducing transmission of the virus in persons already infected with HIV has been identified as an urgent research and public health priority [7,8]. HIV testing and counselling services are considered essential components of a comprehensive HIV prevention program due to the potential to reduce HIV transmission. Several authors [9-11] have reported that high risk behaviour is lower among individuals who are aware of their HIV status, compared to individuals who are unaware. A recent report on HIV testing and counselling [12] highlighted the need for research to explore the structural factors that influence counselling, testing and partner notification, particularly among individuals who are socially marginalized and most vulnerable to infection.

Research on HIV testing among youth suggests that, in general, younger people perceive that they are less vulnerable [2,13] and are less likely than older individuals to access HIV testing [14]. However, youth at higher risk for HIV who are connected with programs may be more likely to test for HIV [15,16]. There is no published literature specifically on HIV testing among Canadian Aboriginal youth despite this population being highly susceptible to infection; however, some evidence suggests that Aboriginal youth access HIV testing as much or more than the general population [17]. Similarly there is little research on HIV care received by Canadian Aboriginal youth, although one study reported that following infection with HIV, youth faced challenges in accessing appropriate services, particularly from AIDS service organizations [18].

Knowledge about HIV testing and care in the Aboriginal community is limited. Research suggests that Aboriginal persons may be reticent to access testing and care for HIV. Ontario Aboriginal inmates were less likely to have ever had an HIV test and more likely to report that they didn't know where to get a test than non-Aboriginal inmates [19]. An analysis of Canadian AIDS cases between 1996 and 2005 revealed that Aboriginal persons were significantly more likely to have a late diagnosis of their HIV infection [20]. This trend also was suggested in an analysis of AIDS-related deaths among First Nations individuals in Vancouver [21]. First Nations individuals became more acutely ill earlier, had shorter survival rates, and more admissions and hospital days than others. A more recent study [22] reported that Aboriginal persons accessing highly active antiretroviral therapy (HAART) had shorter survival rates and mortality rates 3 times higher than non-Aboriginals.

In order to focus on prevention activities, including during the post-infection period, it is important to assess factors that motivate individuals to have an HIV test, and subsequently make decisions about their care and treatment. The purpose of this national community-based study was to answer the following research questions:

• Why do Aboriginal youth have an HIV test? Why do they not have an HIV test?

• What are the testing behaviours of Aboriginal youth and what types of services do they use?

• Among Aboriginal youth, what is the relationship between HIV testing and the decision to initiate treatment?

In this paper we present findings from interviews and surveys related to these key questions. Pseudonyms are used in the quotes from the interviews.

## Methods

In light of the limited knowledge about HIV testing and care in Aboriginal youth in Canada, an exploratory mixed method design was selected, including a cross-sectional survey and in-depth interviews. Eleven community-based organizations in seven provinces and one territory assisted with recruitment of participants between January 2004 and February 2005. The organizations included Aboriginal health and friendship centres and AIDS service organizations. Aboriginal men, women, and transgendered between 15 and 30 years of age, living in rural and urban communities, English or French speaking, were invited to participate. The term *Aboriginal *referred to youth who self-identified as First Nations, Métis, or Inuit.

A community-based research (CBR) approach, incorporating the principles of Ownership, Control, Access and Possession (OCAP) for research involving Aboriginal communities guided the study [23]. The project was a collaboration between the Canadian Aboriginal AIDS Network (CAAN), the Public Health Agency of Canada (PHAC), and researchers at the Universities of Alberta, Calgary, and Toronto. Ethical approvals were obtained from research ethics boards at each university, PHAC, and the Aurora Research Institute in the Northwest Territories. The research team received direction and guidance on the questions, the design of the study, and the interpretation and dissemination of the findings from a community advisory committee (CAC) that included Aboriginal youth, Aboriginal Elders, and representatives from community-based organizations. This overall approach ensured that the research was congruent with the Canadian Institutes for Health Research (CIHR) *Guidelines for Health Research Involving Aboriginal People *[24].

A self-administered survey was developed based on an extensive review of the published literature, unpublished reports, and the experience of research team members. It was pre-tested with the CAC to enhance face and content validity, translated into French, and then was pilot tested with two groups of Aboriginal youth, one English and one French speaking (total n = 19). A representative from each participating organization completed a training session to ensure methods were consistently applied. Convenience and network sampling [25] were used to recruit youth to complete the surveys or participate in an interview. Prior to enrolment, researchers ensured free and informed consent for each participant and obtained necessary signed documentation. Participants were provided with a private space to complete the survey. To ensure confidentiality, the two project coordinators (one for Eastern Canada and one for Western Canada) distributed the surveys, and all participants placed their completed survey in an envelope. Survey and interview respondents were provided with a small token of appreciation for their participation. Participating organizations also received a small compensation for staff time spent promoting the research project and assisting with the recruitment.

### Data Management and Analysis

Survey responses were coded, entered, and verified using SPSS version 11.5 statistical software (SPSS, Inc., Chicago IL.). Descriptive statistics were produced for all survey items, and cross-tabulations and associational statistics were produced for key outcomes including HIV testing. Bivariate relationships were examined using appropriate statistics (e.g., Chi-square, Fisher's Exact test, t-test). A two-sided *p *value of less than 0.05 was considered to indicate statistical significance. Open-ended survey item responses were coded using thematic analysis [26]. The coding framework for the qualitative interviews was developed inductively by three members of the research team, including the principal investigator, one co-investigator, and one project coordinator. This process involved the reading and discussion of three interviews to develop consensus on the emerging themes. The qualitative software program QSR N6 was used to assist with the labelling, revising, and retrieval of codes during analysis of the interviews.

## Results

### Sample

Four hundred and thirteen (n = 413) Aboriginal youth from Vancouver, Edmonton, Winnipeg, Ottawa, Toronto, Montreal, Halifax, Labrador, and Inuvik completed the survey and twenty-eight (n = 28) Aboriginal youth participated in in-depth, semi-structured interviews. Youth from all ten provinces and one territory participated in the study. The survey and interview samples were similar in terms of gender distribution of participants (females: 52.8% in survey, 57.1% in interviews). The survey participants were slightly younger (average age = 21.5 years, median = 21.0 years) than the interview participants (average age = 24.4 years, median = 24.0). Approximately half (50.8%) of the survey participants had tested for HIV (n = 210), and of these, 26 (12.4%) were HIV-positive. In comparison, almost all interview participants (89.2%) had tested for HIV, and of these, 9 (36.0%) youth were positive. Tables 1 and 2 provide additional information about participant characteristics.

**Table 1:** Characteristics of survey participants

Variable^a,b ^n = 413	%
Age (Mean ± SD years) range 15–30 years	21.5 ± 4.4
Gender	
Male	47.0
Female	52.8
Transgender	0.2
Aboriginal background	
First Nations Status	56.2
First Nations Non Status	6.8
Métis	20.8
Inuit	10.7
Inuvaluit	2.2
Other	1.7
Languages spoken by youth	
English	97.8
Aboriginal Language	13.1
French	22.0
Sexual Identification	
Heterosexual or straight	84.7
Gay/lesbian/two-spirit/bisexual	13.1
Other	1.5
Marital status	
Single	69.2
Common-law relationship	20.3
Married	3.6
Separated/divorced/widowed	3.4
Education^c^	
Grade 8 or less	10.7
Some high school	49.9
Completed high school	16.2
College/university	7.8
Some post secondary	14.6
Religion and spirituality	
Followed a religion	41.4
Followed traditional Aboriginal practices	41.0
Place of residence	
Living in urban centre, population > 10,000	64.4
Own home/apartment	35.8
Parents' home	31.0
Friend's home	9.7
Rooming house	3.1
Shelter or safe house	4.4
Hotel	3.4
Homeless	4.6
Other living arrangement	8.2
Income Sources^c^	
Had job	37.3
Social support/welfare	29.3
Several sources of income	16.4
No income	6.5
HIV testing	
Tested for HIV	50.8
Tested positive (among those tested)	12.4

^a ^Certain variables do not add up to 100% as only some sub-variables are shown. Many variables have missing data as participants could choose not to answer the survey questions or answer more than once per question.

^b ^Four hundred and thirteen (413) Aboriginal youth from Vancouver, Edmonton, Winnipeg, Ottawa, Toronto, Montreal, Halifax, Labrador and Inuvik completed the survey.

^c ^As many of these youth were in the process of obtaining an education, it is expected that the reported level of education and personal (not household) income are variable indicators of life circumstances.

**Table 2:** Characteristics of interview participants

Variable n = 28	N
Mean age	24.4 years
Age	
16–19	5
20–24	10
25–30	13
Gender	
Male	12
Female	16
Education	
Grade 8 or less	3
Some high school	9
Completed high school	5
Some post secondary	11
College/university degree	4
HIV testing	
Tested for HIV	25
Tested positive for HIV	9

Of the 28 interview participants, 25 self-identified as First Nations (FN), two were Inuit, and one was Métis. While youth participating in the interviews shared many stories of very challenging circumstances (e.g., street involvement, homelessness, prostitution, sexual abuse as a child, and trouble with the law), many also shared stories that reflected their sense of personal responsibility, focus on personal health, involvement with community, family, and cultural activities.

### Reasons for testing or not testing for HIV

**Figure 1:**
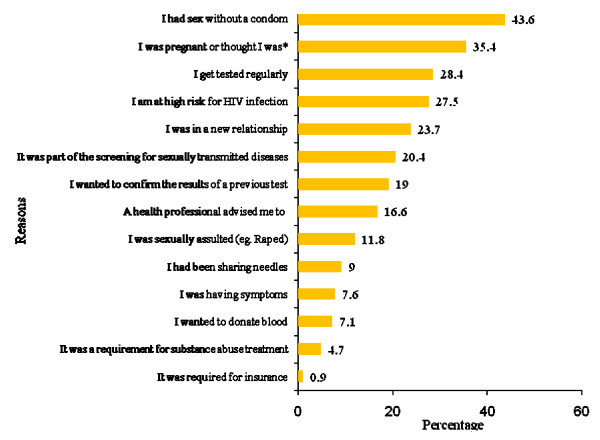
**Reason for HIV testing (n = 210)**. *adjusted to female participants.

Surveyed youth were presented with a list of reasons for HIV testing from which they could choose multiple responses (see Figure 1). Among youth who had had an HIV test (n = 210), the most common reasons for testing were having sex without a condom (43.6%), and being pregnant or thinking that they were (35.4%).

**Figure 2:**
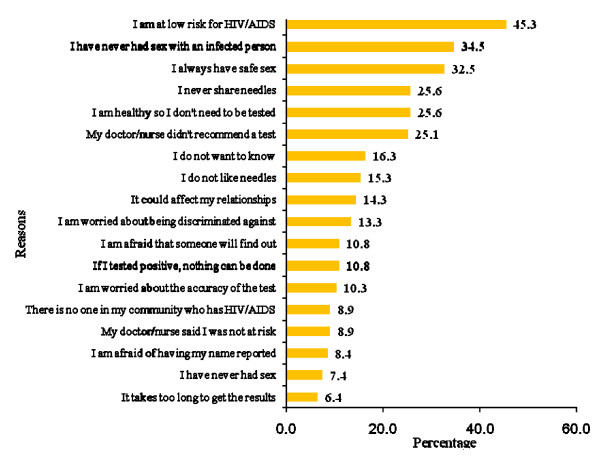
Reasons for not testing for HIV (n = 203).

Youth also were presented with a list of reasons for not testing for HIV (n = 203) (see Figure 2). The most common reasons provided for not getting tested were a self-perception of being a low risk for HIV (45.3%) and the self-perception of not having had sex with an infected person (34.5%).

The youth who participated in interviews shared many attitudes and beliefs related to HIV, AIDS, and sexuality that provide a more in-depth understanding of factors that may influence youths' decision to have an HIV test. For example, the feeling of "invulnerability" in relation to HIV risk and the belief that "it can't happen to me" were common among participants. The youth attributed these feelings to normal "risk-taking" youth behaviour and, at times, a greater concern with issues such as pregnancy:

*But they don't think about HIV or any other sexually transmitted infections or anything. But they think about pregnancies. Just totally blank to their mind about [pause] STIs and AIDS ... That's the last thing they're thinkin' of when they're doin' it. They're thinkin' of 'Am I going to get this girl pregnant?' [laughs] That's the only thing they're thinkin' of*.

(Oliver, First Nations [FN], Male)

*All kids engage in risky behaviour. It's part of being young, is just taking risks and taking chances and being dumb, in some cases. They know that their friends are doing it and have been doing it, and they're fine, and they've not had any problems, no one's gotten pregnant yet or whatever*...

(Irene, FN, Female)

Among the interviewed youth, it was very common to fear HIV, AIDS, and HIV testing and some of the youth viewed AIDS as a death sentence. The fear of AIDS could be both a motivator and a detractor to HIV testing. For example, Sara was positive for hepatitis C, and although she felt that her risk of AIDS was lower, she was very frightened of AIDS and believed that the majority of persons infected with the disease would die:

*I'm afraid of all the STDs altogether. STDs top to bottom. That's all something very frightening. Some of them can be treated, but some of them can't be. And with me already having Hepatitis C, I mean, I just [pause] I'm nervous about all of them. I think AIDS is something that there's a lower risk of me having that, but it is something I'm very, very scared of. With all rights, I should be*.

(Sara, Métis, Female)

Rachel was very fearful of HIV, and her fear of the unknown made her reluctant to go for an HIV test:

*Well, yeah, because if you're not familiar with something, you're going to be afraid of it, right? ... You're going to be scared of doing something like that. That's why I procrastinated on getting HIV testing done. I had to get me pregnant in order to get my HIV test done*.

(Rachel, FN, Female)

In making the decision to have an HIV test, youths' fear of HIV was balanced with a self-assessment of past behaviour that had placed them at risk for HIV. Many youth shared stories of unprotected sexual relationships, street involvement, or injection drug use as factors in their decision to have an HIV test:

*Well, I was wild when I was younger, so I had – I had slept with some people I probably shouldn't have, and it was unprotected, so I'd get really scared, and I'd go get it tested ... because the lifestyle I had was not very safe*.

(Beth, FN, Female)

*I thought if I was HIV-positive, I thought it was better if I found out sooner or later. I guess I was pretty sure that I had it...It was because I used needles, and a lot times, I just didn't care ...And that's the way it gets when you're a drug addict; you just don't care about things like that, right? I just used dirty needles and not give a shit. I done it so many times; you know the majority of people down here that use needles, they have HIV*.

(Trent, FN, Male)

Similar to the survey participants, several of the female interview participants believed that pregnancy was an important reason to get tested for HIV:

*Yeah, I was pregnant. They said you had to get one done, and I'm, like, 'okay.' ... I think they gave me the choice of getting one done, 'cause I think it was optional, but I guess for me, if I was going to have a child, I think I'd want to know. So if I passed it, I know what I was in, at least, you know, give the proper care to my child. So I think what's why I did it. But I didn't want to do it*.

(Paulette, FN, Female)

Some youth commented that topics such as HIV and AIDS and sexuality were taboo in small reserve communities and were not talked about openly. The difficulty that some youth faced in talking to their parents about their own sexuality may have resulted in them being less knowledgeable about HIV, AIDS, and other sexually transmitted infections, and less likely to have an HIV test. Irene had been raised in a large urban area in southern Canada; however, she noticed a different approach to raising children in the northern community where she lived at the time of the interview:

*It's just a different way of life. I mean we can look at it as a Southerner, as someone who's been used to a different culture, one where in school, I was taught about AIDS and HIV and not having sex before a certain age, and not getting pregnant, and stuff like that. Then I came up here and pretty much everyone I met – like young, old, whatever ...I mean everything seems to be different here. Just the way children are raised and what they're taught, it's kind of like they're left to fend for themselves*.

(Irene, FN, Female)

Melanie recalled her mother's discomfort talking about sexuality and related this to her own lack of knowledge about the need for pap smears:

*I didn't know ...Nobody told me. My mom was too scared to tell me ...My mom didn't want to talk to me about that. ...Because most Native youth ain't open with their parents ...I wasn't open with my mother*.

(Melanie, FN, Female)

### HIV Testing Behaviour

Of the 210 youth who had had an HIV test, 205 responded to a question about the test results. Twenty-six (12.7%) indicated they tested HIV positive, 158 (77.0%) said they tested HIV negative, 5 (2.4%) said that their results were not clear and they would have to go back for another test, and 16 (7.8%) said they did not know their HIV status because they had not received their test result. For their most recent HIV test, 154 indicated that they tested in the community where they were currently living or had lived at the time of the test. For the 40 youth who tested outside their current community or the community where they lived at the time of the HIV test, 10 indicated that they did so because "no one knew me," 9 because "I knew the health worker", 6 because "there was no HIV testing in their community", and 3 because "my family was there to support me." The youth who had been tested for HIV were more likely to report a previous STI (47% vs 12%; p < .001), a history of injecting drug use (21% vs 5%; p < .001), or having had anal sex with any partner (25% vs 15%; p < .008) than youth who had not been tested.

**Figure 3:**
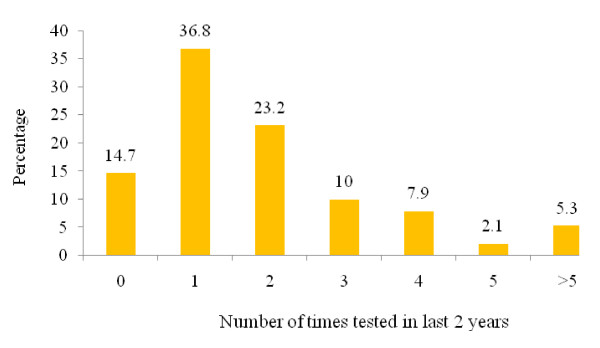
Number of times youth tested within last 2 years (n = 210).

Almost three-quarters (74.1%) of youth who had been tested (n = 210) reported they had taken their last test between 2002 and 2004, and only 13.3% of those who had ever tested for HIV reported that they had not tested within the last two years. The majority of youth (60.0%) had tested once (36.8%) or twice (23.2%) in the past two years (see Figure 3).

The largest percentage of youth (34.1%) had gone to a physician for their most recent HIV test, while another 17.1% had gone to a hospital, 14.6% had gone to a community or public health centre, and 13.7% had attended a walk-in clinic.

The findings from the interview participants support the survey findings. Twenty-five of the 28 interview participants (89.2%) had been tested for HIV and of these, 9 (36.0%) youth were positive. The majority who were not positive had tested within the previous year, with the time since last HIV test ranging from two weeks to six years. Regular HIV testing was viewed as routine, especially when coupled with checks for other STIs or as part of standard medical care when pregnant. One female participant provides insights about her testing patterns:

*Oh, I get tested every 3 months for everything ... Chlamydia, gonorrhea, everything. From bottom to the top, everything ... so I get my HIV testing as well ... I like to get tested regularly. I'm a hypochondriac, so it's, like, you know [laughs], like, to know there's nothing wrong with me*.

(Sara, Métis, Female)

Several of the interview participants considered themselves to be "regular testers". Adam expressed a common reason for testing regularly:

*Now I have a girlfriend that I've been going out with for over a year, and every – once a year, I go and I get tested. I get a physical and I get blood work done and everything, just – because you never know...you know what I mean? There's so many ways to – I just get everything done and out of the way just to keep on top of what's going on with myself*.

(Adam, FN, Male)

The location and services offered at the HIV site was an important consideration for youth when deciding where to go for HIV testing. Interviewed youth often tested in hospital or clinic settings and where physician/nursing services were offered:

*I would probably go directly to the hospital. Last summer, I had it done by a clinic. I got pregnant, and the clinic did it for me*.

(Hazel, Métis, Female)

Although most participants tested at medical clinics and hospitals, several expressed support for drop in testing sites that provide flexibility in access to HIV testing services. Sam was able to access HIV testing in conjunction with other services offered by a drop-in centre:

*You could be here to use the food bank. Nobody's really sure. And people are coming, going in and out so much, suppose you duck into one of these doors and get tested, then nobody's the wiser that that's why you were here*.

(Sam, FN, Male)

### The relationship between testing and care

Of the 26 surveyed youth who reported they were HIV-positive, 8 (30.8%) indicated they had symptoms at the time of diagnosis, including 6 (23.1%) who were told they had AIDS. Twenty-five of the HIV-positive youth provided information on accessing care. Twelve (48.0%) said that they had visited a doctor after HIV diagnosis and 10 youth provided information on the time interval between their HIV diagnosis and when they first saw a doctor (see Table 3). Overall, 8 of these 10 youth (80.0%) had seen a doctor for HIV care assessment within one year of diagnosis. Therefore, in this limited sample, only 38% of HIV-infected Aboriginal youth with available information were assessed for care by a doctor within one year of HIV diagnosis.

**Table 3:** Frequency distribution of time interval between HIV diagnosis and first visit to doctor for HIV care assessment

Time interval	Frequency	Percent
0–3 months	5	50.0
4–6 months	2	20.0
7–12 months	1	10.0
13 months – 2 years	1	10.0
more than 2 years	1	10.0

Total	10	100.0

The nine HIV-positive youth in the interview sample showed similar patterns when accessing care following diagnosis. Most did not mention having symptoms of HIV at the time of HIV testing. However, a few who were active injection drug users (IDUs) reported symptoms as one of their reasons for seeking an HIV test. Zak recalled:

*I knew I was positive ... because of the symptoms I got and because I didn't care. I would share spoons with a person I knew was HIV-positive*.

(Zak, FN, Male)

The finding that a few participants who were IDUs had symptoms of HIV illness at the time of diagnosis, suggests that they were not getting tested for HIV despite their high risk behaviours. None of the interview participants however, reported that they had AIDS at the time of diagnosis.

At the time of the interview, all HIV-positive participants had visited a doctor to discuss HIV treatment options. Most saw their physicians regularly to monitor their health; however, their experiences with health professionals and the length of time between diagnosis and care varied considerably for each participant. Zak, a First Nations male, had been HIV-positive for approximately one and a half years. He had tested positive while in prison and reported that *"after [they told me I was positive] there was a bunch of counsellors that came to see me to help me deal with it*." Zelda also accessed care very quickly after her diagnosis; however, she felt that her doctor's level of knowledge was unacceptable.

*For me, when I was diagnosed, the doctor where I'm from had just said, 'Okay, you're HIV-positive'...And that was all there is ... I had to tell him that I'm pregnant. I should be on some meds. I was the one that told him that my baby needs the meds to reduce the risks. It's, like... 'you should phone [provincial AIDS service provider] or somebody, and talk to them.' He did*...

(Zelda, FN, Female)

It took Sam six months before he was ready to see a physician:

*[Aboriginal organization] gave me a referral ... But that bounced back and forth between answering machines, trying to make the initial appointment for about 6 months ... I didn't really want to go see him, because I didn't want to know my results or my counts or none of those things*.

(Sam, FN, Male)

For Vanessa, it took seven years to contact a health professional to discuss her treatment options.

*That's going good, the doctor, yeah .... [S]he gives me tons of information. I didn't know nothing about care or anything like that, but since I came to [western Canadian city], I need care*.

(Vanessa, FN, Female)

When asked why they did not seek care immediately, interview participants reported: being scared or not wanting to think about their disease; being pre-occupied with drugs or alcohol; not wanting to live; not knowing anything about care.

*When I finally did test positive, it was really hard for me – it was really hard for me not to jump off a bridge ...I was thinking, 'What the hell's the point of living? I'm positive now, and I'm going to die. The world has ended,' and stuff like that ... I was in a daze for quite a while after that. I started drinking more – I didn't want to be sober, 'cause I didn't want to think about my disease*.

(Yvonne, FN, Female)

*[After my diagnosis] I was on a down low ...I wasn't seeing doctors and stuff like that. I was really in a bad situation. I was doing lots of drugs and stuff like that. I didn't know anything about care*.

(Vanessa, FN, Female)

Trent continued to inject drugs and avoid his HIV positive diagnosis until he was admitted to hospital with pneumonia.

*[Drug addicts are] more interested in using drugs than maintaining their health. I've really seen people who are really, really ill, and they don't notice that they're ill, 'cause they're using drugs... That's the way I was too. Before, I got really, really sick, and I ended up in the hospital*...

(Trent, FN, Male)

## Discussion

Several of the interviewed youth shared stories of very challenging circumstances, which while placing them at high risk for HIV infection, must be interpreted within the broader social, political, historical, and economic context. In Canada, many Aboriginal individuals and communities share a continuing history of marginalization and discrimination, and an increased burden of ill-health, arising from the legacy of colonization [27,28]. In order to acknowledge this history and plan effective interventions, it is important to shift the dialogue away from *risk behaviours *to the broader concept of *risk environment *[29,30]. Recognition of the influence of the risk environment on vulnerability to HIV has been documented in Aboriginal youth and women. In a recent Canadian study [31], Aboriginal youth were more aware of the structural factors, including the impact of colonialism that influenced their HIV risk than their non-Aboriginal counterparts. Similarly, Aboriginal women living with HIV highlighted the influence of historical trauma on their response to HIV testing and counselling [32].

HIV testing rates and reasons for testing among youth vary depending on the recruitment settings, the population, and the age of the youth. In the current study, approximately half of the surveyed youth had been tested for HIV, but this proportion is probably not representative of Aboriginal youth in general since some of the recruitment was done in settings that provide HIV testing services. Peralta and colleagues [33] reported that 30% of primarily African American youth between 12 and 24 years had been tested for HIV, with older youth being more likely to test. Similarly, a national survey of South African youth [13] found that older youth were more likely to report having been tested for HIV. In a large American survey of sexually active adolescents 22% had been tested for HIV [16]. Testing rates among youth in high risk situations however, may differ from those in the general population. Among youth 18 to 25 years enrolled in a detoxification program a high proportion (85%) had been tested for HIV, and 74% of youth accepted HIV testing as part of a research study [34].

In the current study the most commonly cited reason (43.6%) for testing was "I had sex without a condom", with smaller proportions of youth stating that they had been tested because it was part of screening for sexually transmitted diseases (20.4%) or it had been recommended by a health professional (16.6%). The finding that a large proportion of the youth independently initiated screening is encouraging and has been reported elsewhere, however the limited influence of health professionals on HIV testing differs in comparison to previous research. Concern that HIV had been contracted through sexual behaviour was a major reason for HIV testing in HIV-positive and HIV-negative American youth [35]. Most youth reported that their decision to have an HIV test was also influenced by a health professional's recommendation. A recent chart review [14] to explore reasons for HIV testing among American youth 13 to 18 years of age found that among those with a documented reason for testing, routine screening was the most common reason for HIV testing, with few adolescents having self-initiated their HIV test.

The reasons that youth gave for not having an HIV test are similar to those given in other studies, however the ordering of reasons both confirms and refutes those given in other studies. Almost half (45.3%) of the youth in the current study stated that "I am at low risk for HIV" as the reason for not having had an HIV test. Similarly, 54% of the youth in a study by Peralta and colleagues [33] felt that they were at low risk for HIV. In Peralta's study, the most common reason (63%) that youth had not been tested was they had never been offered an HIV test, whereas in the current study only 25.1% of the youth mentioned that a doctor or nurse had not recommended an HIV test.

The findings in the current study related to where youth went for their most recent test differs significantly from other research findings. The largest percentage (34.1%) of youth went to a physician for their most recent test, while only a small number (4.4%) went to an anonymous testing clinic. Murphy and colleagues [35] reported that approximately two thirds of their sample of both HIV-positive and HIV-negative American youth were tested at clinics that offered anonymous or confidential testing. This variation may reflect the difference in health systems in Canada and the United States, with Canadian youth feeling more comfortable accessing physicians for HIV testing due to universal health care.

Youth participants were fearful and anxious about HIV, AIDS, and HIV testing and a significant proportion viewed AIDS as a death sentence. The portrayal of HIV and AIDS in Aboriginal media using emotionally charged, often negative terminology [36] may contribute to the fear of HIV and AIDS. Furthermore, HIV and AIDS have been strongly associated with death since early in the epidemic [37], particularly prior to the availability of treatments such as antiretrovirals. MacPhail and colleagues [38] reported that South African youth associated a positive diagnosis with decreased opportunities for the future, including the ability to marry and become a parent. Aboriginal youth in a recent Canadian study [31] were also anxious about HIV and more likely to view AIDS as a death sentence than non-Aboriginal youth. Similarly, a Canadian study [39] that explored the experiences of Aboriginal men and women living with HIV reported that it was common for participants to equate an HIV diagnosis with a death sentence and that about half of the participants had either considered or attempted suicide following their diagnosis. The authors suggested that this was related to a desire to "numb the pain, to help along the inevitable, or to avoid going through what they had seen others experience" (p.159).

The decision to submit to an HIV test may produce anxiety and fear among adults and youth alike. Worthington and Myers [40] suggest that the anxiety associated with HIV testing is complex and is related to risk perceptions, stigma, and the patient-provider power dynamic. The finding that youth often made the decision to have an HIV test based on their assessment of past behaviour has been reported elsewhere in studies with non-Aboriginal youth. An American study [34] reported that for adolescents in a detoxification program their perception of HIV risk might be more important than their reported risk behaviours in their decision to accept an HIV test.

Previous studies have documented that Aboriginal persons are more likely to have a late diagnosis of their HIV infection [20] and less likely to receive optimal anti-retroviral therapy [41]. A troubling finding in the current study was the delay in seeking medical care following diagnosis with HIV. Only about a third (38%) of the HIV-positive surveyed youth had been to a physician during the first year following diagnosis. Similarly, among the interviewed youth, delays in seeking care from a physician ranged from a few months to seven years, thereby limiting prevention and care opportunities. The delay in seeking care following a positive HIV test seen in the current study is markedly different from studies with non-Aboriginal populations. Seventy-seven percent of HIV-positive youth in the charts reviewed by Grant and colleagues [14] accessed care within two months of diagnosis. Flicker and colleagues [18] reported that urban HIV-positive youth had difficulty accessing resources and suggest that urban youth may be "falling through the cracks" (p.308) in terms of meeting their needs for services and support.

The use of a mixed method design in the current study enabled the research team to describe HIV testing behaviours and care decisions in a large sample of Canadian Aboriginal youth, while at the same time develop a more in-depth understanding of factors influencing youths' decision to have an HIV test and access care. In most cases, the findings from the interview data supported and confirmed the findings from the survey data. In all cases, the richness of the qualitative interview findings assisted the researchers to provide a more comprehensive description of the issues related to HIV testing and care in Aboriginal youth. Despite the benefits related to integrating both qualitative and quantitative data collection methods, the study findings must be interpreted carefully in light of the sampling techniques used. Both the survey and interview samples were recruited using convenience and network sampling and weighted toward those who had tested for HIV to reflect the research interest. Therefore, the findings may not be representative of the true frequency of testing among Aboriginal youth in the community. Furthermore, HIV testing experiences and the time period between diagnosis and accessing care were self-reported and therefore response bias may have influenced the youth responses. The findings from the current study provide important new insights into the factors that motivate Aboriginal youth to have an HIV test, and subsequently, if positive, make decisions about accessing care and treatment. This is an important contribution, particularly in light of the limited research on the health needs of Métis, urban Aboriginals, First Nations people not on reserve, and Aboriginal women and children in Canada [42].

Accessible, confidential, and/or anonymous HIV testing is an integral component of a comprehensive approach to HIV prevention. While recognizing the critical importance of HIV testing, it is important to acknowledge that it is not a panacea to eliminate HIV transmission within communities and populations. A few researchers have raised interesting paradoxes related to HIV testing, demonstrating an increase in risk behaviours among HIV testers. Ryder and colleagues [43] reported that in a Canadian population, repeat HIV-negative testing frequently resulted in confusion about what constituted risk and occasionally led individuals to think that they were immune to HIV. Mechoulan [44] used data gathered from American men who had sex with men (MSM) to demonstrate that following the introduction of antiretrovirals, HIV testers took more risks than non-testers. In the current study, a significant proportion (25.2%) of the youth had tested more than twice in the previous two years and some admitted to being *regular tester*. The relationship between HIV testing and behaviour change is complex and therefore, messages encouraging HIV testing in Aboriginal youth must be accompanied by HIV prevention information.

## Conclusion

In the current study, approximately half of the surveyed youth and almost all of the interviewed youth had been tested for HIV. Of the youth who had tested for HIV, 12.7% of the surveyed youth and 36% of the interviewed youth indicated that they were HIV-positive. These rates are likely to be higher than the HIV sero-prevalence rates of Canadian Aboriginal youth due to limitations in the study design described above. The most common reasons for the surveyed youth to have an HIV test were they had had sex without a condom and, for females, were pregnant or thought that they were. Among both groups of youth, the feeling of invulnerability and the perception that they were at low risk for HIV was common. It is encouraging that many of the youth who tested for HIV did so based on a realistic self-assessment of their HIV risk; however, it is of concern that for others, a feeling of invulnerability was a barrier to testing. Furthermore, it is worrying that there frequently was a delay in seeking health services for the youth who tested positive. These findings provide important insights for the design and implementation of comprehensive HIV testing services for Canadian Aboriginal youth that acknowledge the influence of structural factors on HIV vulnerability.

## Competing interests

The authors declare that they have no competing interests.

## Authors' contributions

JM, the principle investigator, and RJ conceptualized the focus for the research project and oversaw the entire research process. RJ and TP liaised with Aboriginal organizations in several centres across Canada to assist with accessing and recruiting the sample. JM, TP and RJ coordinated data collection activities. JM, RJ, CAW, CPA, TW, TM, and TP worked with the community advisory committee to develop the survey tool and provide guidance on the design, analysis, and dissemination process. CAW, CPA, TW, SS and TM provided expertise for the analysis and interpretation of the quantitative data. JM, RJ, and TP provided expertise for the analysis and interpretation of the qualitative data. All authors assisted with the development of the manuscript and read and approved the final manuscript.

## Pre-publication history

The pre-publication history for this paper can be accessed here:


